# Thioterpenoids as Potential Antithrombotic Drugs: Molecular Docking, Antiaggregant, Anticoagulant and Antioxidant Activities

**DOI:** 10.3390/biom12111599

**Published:** 2022-10-30

**Authors:** Alexander A. Ksenofontov, Pavel S. Bocharov, Elena V. Antina, Oksana G. Shevchenko, Aleksandr V. Samorodov, Ilmir R. Gilfanov, Roman S. Pavelyev, Olga V. Ostolopovskaya, Valeriya A. Startseva, Inna V. Fedyunina, Zulfiya R. Azizova, Salavat I. Gaysin, Svetlana V. Pestova, Evgeniy S. Izmest’ev, Svetlana A. Rubtsova, Mohammed A. Khelkhal, Liliya E. Nikitina

**Affiliations:** 1 G.A. Krestov Institute of Solution Chemistry of the Russian Academy of Sciences, 1 Akademicheskaya Street, 153045 Ivanovo, Russia; 2 Biologically Active Terpenoids Laboratory, Institute of Fundamental Medicine and Biology, Kazan Federal University, 18 Kremlevskaya Street, 420008 Kazan, Russia; 3 Center of Collective Usage «Molecular Biology», Institute of Biology, Komi Science Centre, Ural Branch of Russian Academy of Sciences, 28 Kommunisticheskaya Street, 167982 Syktyvkar, Russia; 4 Department of Pharmacology, Bashkir State Medical University, 3 Lenina str., 450008 Ufa, Russia; 5 Varnishes and Paints Department, Kazan National Research Technological University, 68 K. Marksa Street, 420015 Kazan, Russia; 6 General and Organic Chemistry Department, Kazan State Medical University, 49 Butlerova Street, 420012 Kazan, Russia; 7 Medical Chemistry Laboratory, Institute of Chemistry, Komi Scientific Centre, Ural Branch of Russian Academy of Sciences, 48 Pervomaiskaya Street, 167000 Syktyvkar, Russia

**Keywords:** *S*-containing monoterpenoids, molecular docking, P2Y_12_ receptor, antioxidant activity, oxidative hemolysis, antiaggregant and anticoagulant activities

## Abstract

Natural monoterpenes and their derivatives are widely considered as effective ingredients for the design and production of new biologically active compounds with high antioxidant, antimicrobial and anti-protozoa properties. In this study, we synthesized two series of thiotherpenoids “sulfide-sulfoxide-sulfone”, with different bicyclic monoterpene skeleton (bornane and pinane) structures. The effect of the obtained compounds on platelet aggregation was investigated by using the molecular docking technique. The obtained data revealed that all the synthesized compounds may act as potential inhibitors of platelet aggregation. Moreover, the studied sulfides have shown high antioxidant activity as revealed by lipid peroxidation (LPO) process inhibition in a non-cellular substrate containing animal lipids. The sulfides were able to inhibit erythrocyte oxidative hemolysis, to reduce the accumulation of secondary LPO products in cells and to prevent the oxidation of native oxyhemoglobin. Additionally, the corresponding sulfones and sulfoxides exhibited insignificant antioxidant activity. However, the sulfides were found to exhibit significant antiaggregant and anticoagulant effects. These findings suggest as well that the sulfides could serve as a leader compound for future research and possible practical applications.

## 1. Introduction

It is well-known that both natural monoterpenes and their synthetic sulfur-containing derivatives enjoy diverse pharmacological properties [1,2]. Previously, we developed synthetic approaches for obtaining sulfur-containing monoterpenoids of different structures [3]. The obtained compounds exhibited antifungal, anti-inflammatory, antimicrobial, antihelicobacter, antiaggregant and anticoagulant activities.

Recently, we have shown that sulfur-containing monoterpenoids, to a greater extent than oxygen and nitrogen-containing analogues, can reduce spontaneous and induced platelet aggregation [4]. Moreover, it was shown that all the studied monoterpenoid compounds were able to suppress the platelet aggregation in vitro apparently via blocking their P2Y_12_ receptor activity. Molecular docking indicated that the binding force with platelet P2Y_12_ receptor depends strongly on the contained heteroatom in the structure of monoterpenoids, where it was found stronger in the presence of sulfur and decreased in the presence of oxygen and nitrogen atoms, respectively. On the other hand, detailed NMR studies on dodecyl phosphocholine (DPC) as the membrane model revealed that only *S*-containing compound binds to DPC micelles surface. This binding reinforces the mechanical properties of the cell membranes and prevents destabilizing and the following clot formation on the phospholipid surface. However, no confirmation of stable complex formation between DPC micelles and *O*- and *N*-containing compounds were obtained from solution state NMR data. Most likely, for all the studied compounds, both mechanisms of action are realized—receptor and membrane factors—but in the case of the *S*-containing compound, both factors were more pronounced. On a wider lever, considering the low toxicity, the ability to block induced-aggregation, *S*-containing monoterpenoids seem to be promising agents for blood product stabilization, treatment, and the prevention of thrombophilia.

Cellular damage arising from an imbalance between free-radical generating and scavenging systems (“oxidative stress”) has been implicated in the pathogenesis of a wide range of human disorders, including the ischemia-reperfusion injuries characteristic of neurological disorders, such as strokes. Many reports demonstrated that the antioxidative activity of terpenoids may be related to their relaxing effect on the vascular wall and action as antagonists of platelet-activating factor, which improves blood flow and has a stimulating effect on the secretion of neurotransmitters [5].

**Figure 1:**
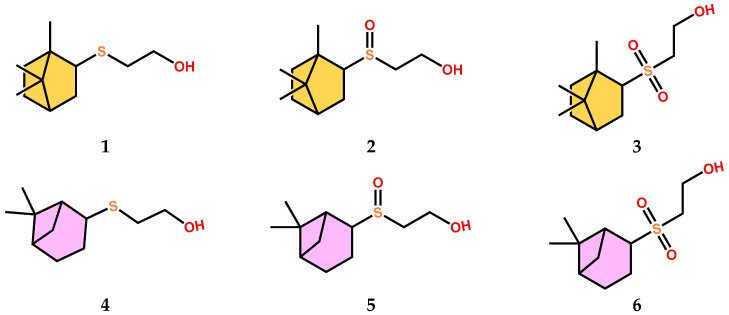
Structures of compounds **1–6**.

In this work, we synthesized two series of thioterpenoids “sulfide-sulfoxide-sulfone”, differing from each other in the structure of the terpenic skeleton. The obtained compounds **1–3** present bornane series compounds, while compounds **4–6** present the pinane series (Figure 1). It was important for us to establish the dependence of the antiaggregant properties of the molecules both on the degree of oxidation of the sulfur atom and on the structure of the terpenic skeleton.

Synthesis, physicochemical properties, and spectral data of compounds **1–6** were described in detail in our previous papers [6,7,8,9].

**Scheme 1:**
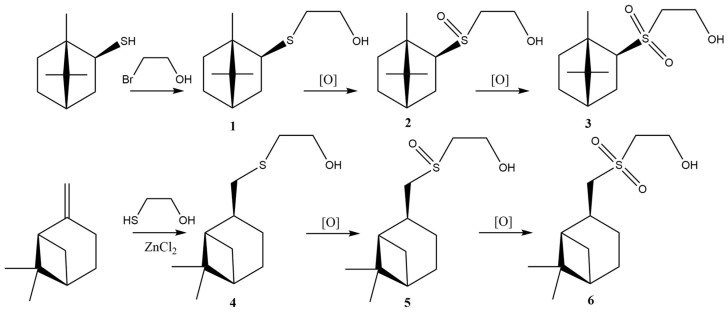
Synthesis of compounds **1–6**.

Reaction of isobornanethiol with 2-bromoethanol in the presence of Cs_2_CO_3_ and tetrabutylammonium iodide afforded a 90% yield of sulfide **1**. Controlled oxidation of **1** with the tert-butyl hydroperoxide-[VO(acac)_2_] system led to sulfoxide **2**. Subsequent oxidation of **2** with the same tert-butyl hydroperoxide-[VO(acac)_2_] system afforded sulfone **3** (Scheme 1) **[6]**. Previously, we performed the addition of 2-mercaptoethanol to the double bond of (1S)-(–)-β-pinene in the presence of zinc chloride resulting in the sulfide **4 [7]**. Sulfoxide **5** and sulfone **6** were synthesized in according to the same procedure described for compounds **2** and **3 [6]**. It should be noted that both sulfoxides **2** and **5** were obtained in racemic form in terms of the configuration of the sulfoxide group.

We have evaluated the affinity of thioterpenoids **1–6** to the P2Y_12_ receptor using molecular docking, studied their antioxidant activity, as well as the antiaggregant and anticoagulant activity of **1** and **4** sulfides as the most promising compounds of multifactorial action.

## 2. Materials and Methods

### 2.1. Synthesis

#### 2.1.1. Sulfide **1**

To a solution of isobornanthiol 1 (221 mg, 1.3 mmol) in ethanol (3 mL), Cs_2_CO_3_ (424 mg, 1.3 mmol) and tetrabutylammonium iodide (480 mg, 1.3 mmol) were added under argon atmosphere. After 5 min stirring, 2-bromoethanol (71 mL, 1 mmol) was added dropwise, then the reaction mixture was refluxed for 24 h. After filtration, the clear solution was concentrated in vacuo, and the residue was purified by silica gel column chromatography using petroleum ether-EtOAc (1:1) as eluent. Sulfide **1** was obtained as a liquid in 90% yield [α^20^ D] = +248 (c = 0.3, chloroform).

#### 2.1.2. Sulfide **4**

A solution of (−)-β-pinene (0.015 M) in CH_2_Cl_2_ was treated at room temperature and stirred with mercaptoethanol (0.0195 M) in CH_2_Cl_2_ (20 mL) and ZnCl_2_ (0.2 g). After 2 h the reaction mixture was treated with water (200 mL), extracted with CH_2_Cl_2_ and dried over MgSO_4_. After solvent was removed, the reaction products were purified by column chromatography over silica gel (hexane:ether). Sulfide **4** was obtained as a liquid in 84% yield.

#### 2.1.3. General Procedure for Sulfoxides **2** and **5** Synthesis

The catalyst VO(acac)_2_ (26.7 mg, 0.1 mmol) was added to a solution of **1** or **4** (214 mg, 1 mmol) in CHCl_3_ (5 mL). After 5 min stirring, a 40% solution of TBHP in CHCl_3_ (0.56 mL, 2.5 mmol) was added dropwise and the reaction mixture was stirred at room temperature for 3 h. A 2:1 mixture of FeSO_4_ and citric acid saturated aqueous solutions (2 mL) was poured in and the resulting solution was stirred for 15 min, then extracted with CHCl_3_ (20 mL). The organic phase was dried over anhydrous Na_2_SO_4_, filtered and concentrated in vacuo. The residue was purified by silica gel column chromatography (eluent: EtOAc/i-PrOH, 3:1) to give sulfoxides **2** or **5** as a solid in 87–90% yield.

#### 2.1.4. General Procedure for Sulfones **3** and **6** Synthesis

The catalyst VO(acac)_2_ (26.7 mg, 0.1 mmol) was added to sulfoxide **2** or **5** (230 mg, 1 mmol) dissolved in CHCl_3_ (5 mL). After 3 min stirring, a 40% solution of TBHP in CHCl_3_ (0.68 mL, 3 mmol) was added dropwise and the reaction mixture was stirred at room temperature for 2 h. A 2:1 mixture of FeSO_4_ and citric acid saturated aqueous solutions (5 mL) was poured in and the resulting solution was stirred for 20 min, then extracted with CHCl_3_ (20 mL). The organic phase was dried over anhydrous Na_2_SO_4_, filtered and concentrated in vacuo. The residue was purified by silica gel column chromatography (eluent: petroleum ether/EtOAc, 3:1) to give the sulfone **3** or **6** as solid in 95% yield.

### 2.2. Molecular Docking

The molecular docking procedure was divided into two steps. In the first step, we performed blind docking to predict **1–6** localization in the P2Y_12_ receptor. Blind docking was carried out via AutoDock4.2 software by using the Lamarckian genetic algorithm [10]. Receptors were considered rigid molecules; ligand molecules were considered flexible. The structure of P2Y_12_ was taken from the protein database (4ntj) (www.rcsb.org, accessed date: 2 December 2013). The P2Y_12_ receptor structure preparation for molecular docking consisted of several parts: (1) all ligands 6-{4-[(benzylsulfonyl)carbamoyl] piperdin-1-yl}-5-cyano-2-methylpyridine-3-carboxylate, cholesterol, (2R)-2,3-dihydroxypropyl (9Z)-octadec-9-enoate) and water from protein were delated; (2) hydrogen atoms without further structure optimization were added; (3) P2Y_12_ and ligand partial atomic charges were calculated by means of the Gasteiger partial charge calculation method. All steps were carried out via python molecule viewer. Blind docking calculations were performed for a 90 Å × 126 Å × 120 Å grid with the P2Y_12_ molecule in the center and a step of 0.700 Å. Each docking experiment was derived from 50 separate runs that were supposed to end after 25 million energy evaluations. The terpenoid-P2Y_12_ complexes were sorted into groups according to RMSE (standard deviation of atomic positions between this conformation and the cluster reference). For each complex, the conformation with the lowest energy was considered the most stable. Molecular plots were made via UCSF Chimera [11].

In the second step, specified molecular docking via GOLD was performed to refine the binding sites and energy parameters. The most probable ligand location inside the P2Y_12_ pocket from blind docking calculations was chosen as initial calculation point. All atoms within a radius of 20 Å from the ligand were selected. For all ligands 50 separate runs were performed as in the case of blind docking. GoldScore with rescore by ChemScore was chosen as a function to evaluate binding efficiency. In the GA settings, the highest search efficiency of 200% was chosen to get the most accurate results [12].

### 2.3. Biochemical Assays

#### 2.3.1. TBARS Assay

The antioxidant activity of compounds **1–6** was evaluated (in vitro) as inhibition of accumulation of secondary lipid peroxidation (LPO) products in mouse brain homogenates [13,14]. The brain was homogenized in physiological saline (pH 7.4) (10% *v*/*v*) and centrifuged at 3000 rpm for 10 min. The low-speed supernatant containing lipids was separated [13,15]. The test compounds were added to the supernatant in the form of acetone solutions at final concentrations of 0.1 and 1 mM. Then, in 30 min, LPO was initiated by adding a freshly prepared solution of FeCl_2_ and ascorbic acid [15,16]. Samples were stirred gently for 1 h at 37 °C in a thermostated Biosan ES-20 shaker (Latvia). The concentration of secondary LPO products reacting with TBA (TBA-reactive substance, TBA-RS) was determined using a Thermo Spectronic Genesys 20 instrument (USA) at λ 532 nm, with the extinction coefficient of 1.56 × 10^5^ M^−1^·cm^−1^ [14,17,18].

#### 2.3.2. Mouse RBC Test for Erythrotoxicity, Antioxidant and Membrane-Protective Activities

The erythrotoxicity, antioxidant and membrane-protective activities of compounds **1–6** were evaluated in 0.5% (*v*/*v*) suspension of mice RBCs in phosphate-buffered saline (pH 7.4). Erythrotoxicity was assessed by RBC hemolysis after 1–5 h of incubation with the test compounds (final concentration 0.1 mM). Antioxidant and membrane-protective activities were determined by inhibition of induced hemolysis, inhibition of LPO products accumulation (TBA-RS), and oxidation of oxyhemoglobin in RBCs. After the addition of the test compound solutions (100 µM final concentration), the suspension of RBCs was incubated for 30 min, and then hemolysis was initiated by the addition of H_2_O_2_ (final concentration 1.8 mM) or AAPH (2,2′-Azobis(2-amidinopropane) dihydrochloride, 3 mM). The reaction mixture was shaken gently for 5 h at 37 °C in a thermostated Biosan ES-20 shaker (Riga, Latvia). An aliquot was taken from the incubation medium each hour and centrifuged for 5 min at 3000 rpm (1600× *g*). Hemolysis was determined as hemoglobin content in the supernatant at λ 524 nm [19] using a Thermo Spectronic Genesys 20 instrument (East Lyme, CT, USA). The percentage of hemolysis was calculated relative to complete hemolysis of the sample, where it was triggered by addition of distilled water. The concentration of secondary LPO products in the RBC hemolysate was assayed as described above. To assess the accumulation of hemoglobin oxidation products, we have analyzed the absorption spectrum of the hemolysate at λ values of 540–640 nm on a Fluorat-02-Panorama spectrofluorimeter (St. Petersburg, Russia). The contents of oxyhemoglobin (oxyHb), methemoglobin (metHb) and ferrylhemoglobin (ferrylHB) were calculated using corresponding extinction coefficients [20].

Each experiment was repeated 3–8 times. Statistical analysis was conducted using Microsoft Office Excel 2007 and Statistica 6.0 software packages. The data represent the mean ± SE. The statistical significance of the differences was assessed by the Mann–Whitney U test. The critical significance level *p* for statistical criteria was set to 0.05.

The experiments were conducted on the equipment of the Collective Usage “Molecular Biology” Center, Institute of Biology, Komi Scientific Center, Ural Branch of RAS. We used the RBCs mass and brain tissue of intact laboratory mice obtained from the scientific collection of experimental animals at the Institute of Biology, Komi Scientific Center, Ural Branch of the RAS, and registered as a unique scientific installation of the scientific and technological infrastructure of the Russian Federation (http://www.ckp-rf.ru/usu/471933/ accessed on 24 January 2017). The animals were handled under the ‘Regulations on the vivarium of experimental animals’ (protocol no. 1) considering sanitary–hygienic and bioethical aspects.

#### 2.3.3. Antiaggregant and Anticoagulant Activities

The experiments were performed in compliance with the requirements of Good Laboratory Practice Rules of the Eurasian Economic Union in the field of medicine circulation.

Antiaggregant and anticoagulant activities were assessed under in vitro conditions in isolated blood fractions from 24 healthy male volunteers of 18–24 years old. The research was approved by the Ethics Committee of BSMU, Ministry of Health of Russia (No. 1 dated Feb. 20, 2019). Informed consent was obtained from all participants before collecting blood.

The influence of the compounds on platelet aggregation was studied by means of the Born method [21] on an AT-02 aggregometer (SPC Medtekh, Moscow, Russia). The antiaggregant activities of the tested compounds and the reference drug were assessed at a final concentration of 1 × 10^–3^ M with incubation for 5 min. Adenosine diphosphate (ADP) at a concentration of 2 × 10^−5^ M and collagen at a concentration of 5 µg/mL (Tekhnologiya-Standart, Moscow, Russia) were used as aggregation inductors. The reference drug was acetylsalicylic acid (powder substance; Shandong Xinhua Pharmaceutical Co., Ltd., Zibo, China).

The anticoagulant activity was determined by clotting tests [22] in a Solar CGL2110 turbidimetric hemocoagulometer (ZAO SOLAR, Minsk, Belarus). The final concentration of the tested compounds and the reference drug (sodium heparin) was 5 × 10^−4^ g/mL. The activated partial thromboplastin time (APTT), prothrombin time (PT), and fibrinogen concentration according to Clauss were studied. The reference drug was heparin sodium (5000 IU/mL solution for injection, OAO Sintez, Saint-Petersburg, Russia).

Statistical analysis was performed by the Statistica 10.0 software (StatSoft Inc., Tulsa, OK, USA). A normal distribution check was achieved by the Shapiro–Wilk criterion. Variational series were described by calculating the median, 25 and 75 percentiles. The Kruskal–Wallis test (the dataset did not obey normal distribution laws; A-criterion) was performed. The critical significance level *p* for statistical criteria was set to 0.05.

## 3. Results

### 3.1. Molecular Docking Study

P2Y_12_ are purinergic ADP receptors that belong to the G_i_ class of the G-protein-coupled group (GPCR) [23]. P2Y_12_ receptors are located in the platelet membrane and are an important regulator of platelet aggregation [24]. Therefore, we investigated the potential of compounds **1–6** to act as inhibitors of platelet aggregation by evaluating their interaction with the P2Y_12_ receptor protein. As mentioned earlier, terpenoids **1–6** differ from each other in the functional group and the structure of the terpenic skeleton (Figure 1). It is worthy to note that we have previously shown that compound **2** is characterized by two stable conformers [6], while compound **3** can exist in the form of a dimer [6]. These structural features were considered when performing molecular docking: (1) In the case of compound **2**, molecular docking was performed for both conformers (conformer A and conformer B). (2) In the case of compound **3**, computer simulations were carried out for both monomers that make up the dimer (monomer A and monomer B).

**Figure 2:**
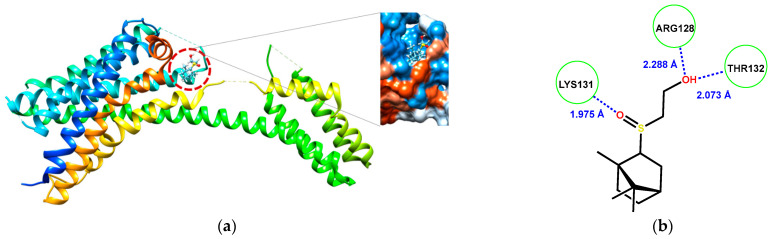
Results of blind molecular docking for the interaction of compound 2 (conformer B) with P2Y_12_. Inset: image of the electrostatic surface of P2Y_12_ with bound compound 2 (conformer B) (**a**). Schematic representation of the interaction of compound 2 (conformer B) with the amino acid residues of the binding site in P2Y_12_ (**b**).

The results of blind docking showed that both groups of terpenoids are localized near the intracellular C-terminal region of the P2Y_12_ receptor protein regardless of their structural features (Figure 2, Figure S1). Analysis of the amino acid residue composition of the hydrophobic cavity, which are located in terpenoids **1–6**, showed that all terpenoids **1–6** are localized in a structurally identical cavity (Table S1).

Terpenoids **1–6** are retained in the P2Y_12_ pocket by van der Waals and hydrophobic interactions as well as the hydrogen bonds to the reactive groups of amino acid residues (Figure 2b, Figure S2). The sites and type of interactions of compounds **1–6** with the reactive groups of amino acid residues are shown in Figure S3. As can be seen from Figure S3, all structures are characterized by: (1) the formation of hydrogen bonds through the interaction of atoms of OH-, S=O- and O=S=O-groups with the functional groups of THR132, LYS131, PHE130, PRO129, ARG128, THR126, LYS125 (1.881–2.499 Å); (2) van der Waals interactions with LEU301, LYS237, VAL234, LYS131, PRO129, ARG128, THR126, LYS125, ARG122; (3) hydrophobic interactions of the atoms of the terpene fragment and the sulfur atom of the sulfide group with LEU301, VAL238, LYS233, PHE130, LYS125, ARG122. Thus, the modification of functional groups and the structure of the terpene skeleton presented by us does not significantly affect the hydrophobicity, and, consequently, the affinity for the discovered P2Y_12_ pocket of compounds **1–6**.

**Figure 3:**
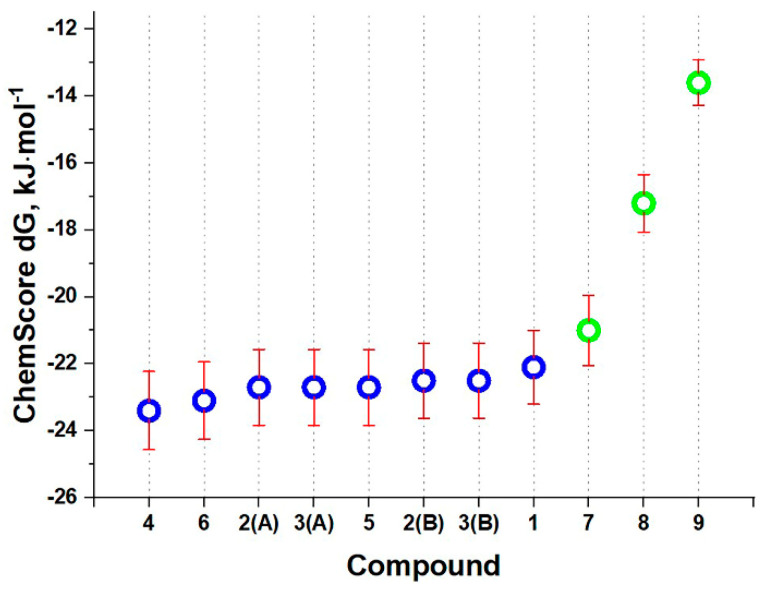
The ChemScore d*G* for «terpenoid–P2Y_12_» system (**2(A)** and **2(B)** refers to **2** conformer A and conformer B respectively; **3(A)** and **3(B)** refers to 3 monomer A and monomer B, respectively (blue circle). The ChemScore d*G* for structurally related terpenoids presented in ref. [4] (green circle).

To evaluate the affinity of terpenoids **1–6** for P2Y_12_, we analyzed the binding free energies (Figure 3). ChemScore d*G* analysis showed that all the structural features of terpenoids **1–6** presented in this article do not significantly affect the binding energy to P2Y_12_ (Figure 3, blue circle). Moreover, the compounds **1–6** were found to have a higher affinity for the P2Y_12_ receptor compared to the structurally related compounds described in our previous work (Figure 3, green circle) [4]. In addition, regardless of the terpene core nature, sulfo-substituted terpenoids (compounds **1–6** and **7** [5]) have the highest affinity to the P2Y_12_ receptor.

It should be noted that we previously carried out the molecular docking procedure for P2Y_12_–ticagrelor AM and P2Y_12_–clopidogrel active metabolite systems [5]. It was shown that clopidogrel active metabolite is localized near the C-terminal region of the P2Y_12_ receptor (binding energy is −28.6 kJ·mol^−1^). Ticagrelor AM is localized near the extracellular *N*-terminal region of the P2Y_12_ receptor (binding energy is −40.1 kJ·mol^−1^). The binding free energy values and the similarity of the amino acid binding site of clopidogrel active metabolite and compounds **1–6** for the P2Y_12_ receptor systems may indicate similar antithrombotic action mechanisms.

Our results demonstrated that the structural and energetic features of the interaction of compounds **1–6** with P2Y_12_ points towards their potential ability to act as inhibitors of platelet aggregation.

### 3.2. Antioxidant Activity

Terpenes are playing a considerable role in the incessant research for new bioactive natural products against oxidation and inflammation because of their unique properties. Some monoterpenes possess both anti-inflammatory and antioxidant properties [25,26]. (+)-Limonene and 1,8-cineole demonstrated strong antioxidant and anti-inflammatory properties. Previous works have demonstrated that the antioxidant and pro-oxidant behavior of a particular terpene depend mainly on its amount. At high concentrations, terpenes can act as pro-oxidant compounds whereas at low concentrations, they can act as antioxidant compounds [27].

In our previous work we studied the membrane-protective and antioxidant activities of terpenic alcohols of pinane, carane and camphane structures with one or two hydroxyl groups [28]. The high antioxidant activity was found for pinane alcohols: on the model of ascorbate-dependent LPO—for (−)-nopol and (−)-cis-verbenol; on the model of oxidative hemolysis initiated by H_2_O_2_ or AAPH— for (−)-cis-verbenol and (−)-myrtenol.

#### 3.2.1. Non-Cellular Model (Antioxidant Activity in a Substrate Containing Animal Brain Lipids)

**Figure 4:**
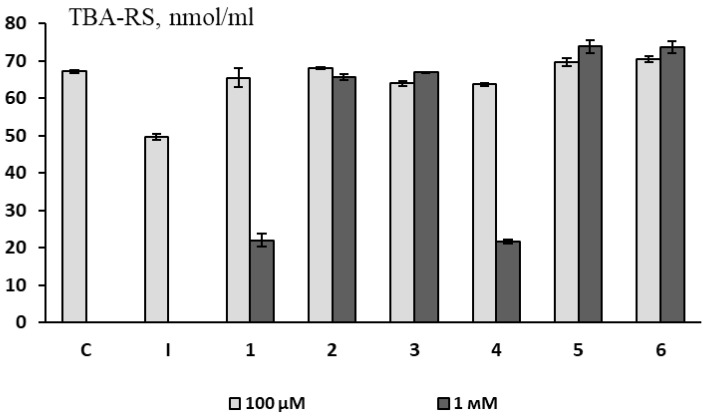
Effects of compounds **1–6** (100 µM and 1 mM) on TBA-RS accumulation in mouse brain homogenates. TBA-RS concentrations were measured at 1 h after the initiation of LPO by Fe^2+^/ascorbate. C—control sample containing no test compounds; I—intact sample in which LPO was not initiated. Vertical bars: means ± SE (*n* = 4–8).

It was shown that sulfides **1** and **4** statistically significantly inhibited both spontaneous and induced LPO in a substrate containing easily oxidized animal lipids at a concentration of 1 mM (level of statistical significance, *p* = 0.006 for compound **1** and 0.004, for compound **4**, respectively) (Figure 4). Sulfides **1** and **4** were close to the previously studied (−)-cis-verbenol in terms of their ability to inhibit the accumulation of LPO secondary products (TBA-RS) and surpassed most of the other studied bicyclic monoterpene alcohols ((−)-neoisoverbanol, (+)-3α, 4α-carandiol, (−)-3β, 4α-carandiol, (+)-3β, 4β-carandiol, (−)-2α, 3α-pinandiol, (−)-2α, 3β-pinandiol, (−)-3α, 4β-pinandiol, pinocampheol, (±)-2-exo-10-endo-camphandiol, (−)-isocaranol-4, (−)-cis-myrtanol, (−)-trans-myrtanol, (−)-myrtenol) [28]. However, sulfides **1** and **4** at a concentration of 1 mM were less active than trolox, an analogue of vitamin E [29,30]. For sulfoxides **2** and **5**, as well as for sulfones **3** and **6**, no antioxidant activity was detected in this model system.

#### 3.2.2. Mouse RBC Test for Erythrotoxicity, Antioxidant and Membrane-Protective Activities

**Figure 5:**
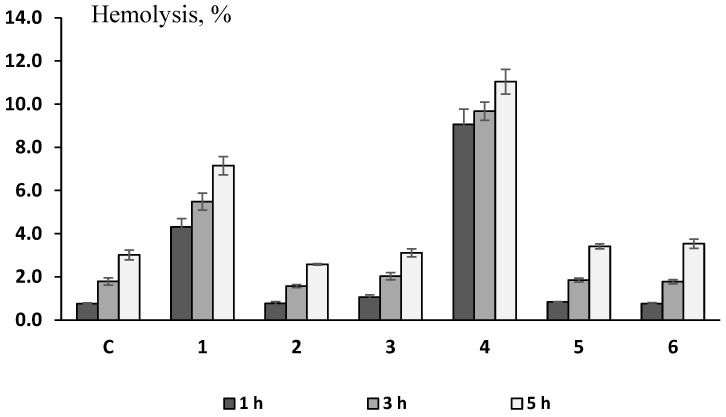
Hemolytic activity (erythrotoxicity) of compounds **1–6** (100 µM) after 1, 3 and 5 h of erythrocytes incubation. Vertical bars: means ± SE (*n* = 3–6).

Among compounds **1–6** at a concentration of 100 µM, sulfides **1** and **4** showed the highest hemolytic activity (Figure 5). Furthermore, the erythrocyte survival rate in the presence of these compounds was never less than 89%, suggesting the possibility of further studies of the activity of these compounds at this concentration.

**Figure 6:**
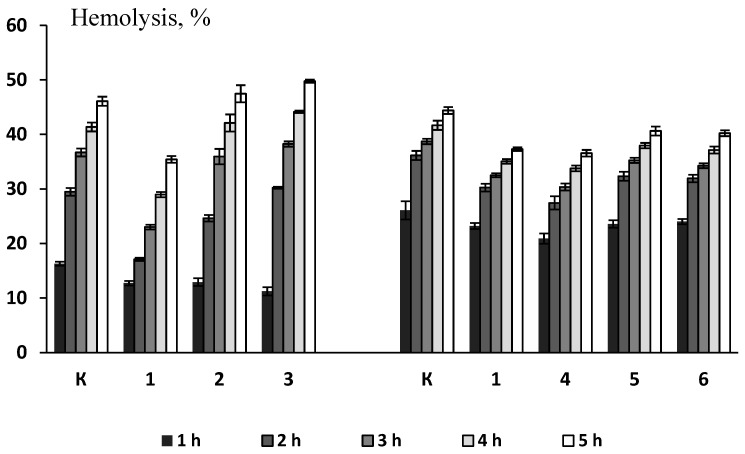
The influence of compounds **1–6** (100 µM) on the level of H_2_O_2_-induced erythrocyte hemolysis after 1–5 h of incubation. Vertical bars: means ± SE (*n* = 3–6).

**Figure 7:**
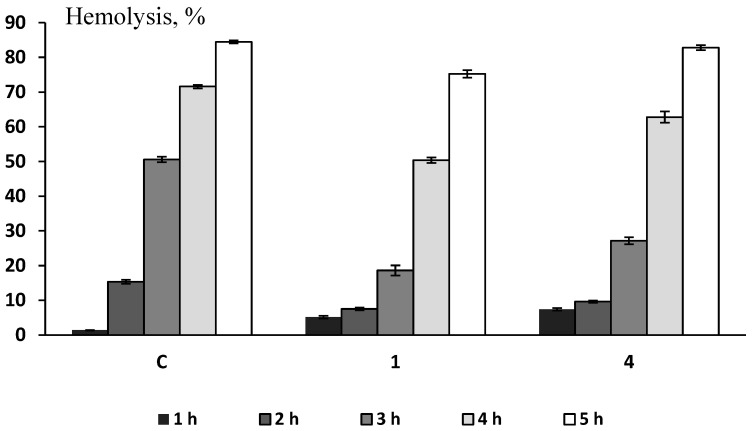
The influence of compounds **1** and **4** (100 µM) on the level of AAPH-induced erythrocyte hemolysis after 1–5 h of incubation. Vertical bars: means ± SE (*n* = 3–6).

Sulfides **1** and **4** can not only inhibit LPO in a non-cellular substrate (Figure 4) but also statistically significantly protect red blood cells from death under conditions of acute oxidative stress induced by H_2_O_2_ (*p* = 0.001, compound **1** and 0.007 compound **4**, respectively) (Figure 6) or AAPH (*p* = 0.004 compound **1** and 0.004 compound **4**, respectively) (Figure 7). In general, they statistically significantly reduce the accumulation of secondary LPO products and prevent the oxidation of native oxyhemoglobin in cells more actively than sulfones and sulfoxides (Table 1). Trolox at the same concentration under conditions of H_2_O_2_-induced oxidative stress exhibited relatively low activity [31] despite the high radical scavenging activity in the DPPH test.

### 3.3. Antiaggregant and Anticoagulant Activities

According to the obtained results, we suggest that sulfides **1** and **4** could serve as leader compounds for future research. Thus, we have determined their indicators of platelet aggregation and plasma hemostasis (Table 2).

The obtained data from the comparison of the effect of compounds **1**, **4** and commercial drugs on platelet aggregation induced by ADP showed that compound **1** exhibits antiaggregatory activity exceeding the values of acetylsalicylic acid by 1.9 times (*p* < 0.001), and compound **4** exhibits antiaggregatory activity at the level of acetylsalicylic acid. At the same time, the studied compounds significantly shortened the latency period in comparison with the control and the comparison drug.

Additionally, compounds **1** and **4** caused hypocoagulation, increasing APTT by 5.6% and 9.2%, respectively, compared with the control sample, and did not affect the concentration of fibrinogen and prothrombin time. The evidence of the effect of the studied compounds was significantly inferior to the effect of heparin, which increased APTT by 20.3%.

## 4. Conclusions

Using the molecular docking procedure, we have shown that compounds **1–6** can act as potential inhibitors of platelet aggregation. Moreover, the evidence from this study suggests that sulfides **1** and **4** exhibit antioxidant activity, manifesting in the ability to inhibit LPO processes in a non-cellular substrate containing animal lipids, to inhibit H_2_O_2_ and AAPH-induced erythrocyte hemolysis. This has been found to reduce the accumulation of secondary LPO products in cells and prevent oxidation in native oxyhemoglobin. Additionally, the corresponding sulfones and sulfoxides were found to exhibit insignificant or no antioxidant activity. What is more, our results confirmed the significant antiaggregant and anticoagulant effects of compounds **1** and **4**. These findings suggest as well that sulfides **1** and **4** could serve as a leader compound for future research and possible practical applications.

## Figures and Tables

**Table 1:** The influence of compounds **1–6** (100 µM) on the parameters of H_2_O_2_ and AAPH-induced hemolysis of red blood cells. * *p* ≤ 0.05, ** *p* ≤ 0.001–in comparison with the control.

Experiment 1 (H_2_O_2_-Induced Hemolysis)			
Control	2.14 ± 0.05	1.03 ± 0.07	0.87 ± 0.03
1	1.59 ± 0.06 **	0.99 ± 0.06	0.72 ± 0.03 *
2	1.87 ± 0.05 **	1.34 ± 0.11	0.86 ± 0.04
3	1.85 ± 0.03 **	1.65 ± 0.10	0.83 ± 0.05
Experiment 2 (H_2_O_2_-induced hemolysis)			
Control	2.34 ± 0.04	1.14 ± 0.06	0.88 ± 0.04
1	2.08 ± 0.04 **	0.98 ± 0.06 *	0.66 ± 0.02 **
4	1.82 ± 0.03 **	0.94 ± 0.10 *	0.59 ± 0.03 **
5	2.07 ± 0.06 *	1.77 ± 0.12	0.77 ± 0.05
6	2.11 ± 0.04 *	1.51 ± 0.07	0.65 ± 0.04 *
Experiment 3 (AAPH-induced hemolysis)			
Control	2.17 ± 0.04	2.85 ± 0.13	1.67 ± 0.10
1	1.69 ± 0.04 **	1.90 ± 0.19 **	1.00 ± 0.05 **
4	2.03 ± 0.06	3.38 ± 0.28	1.59 ± 0.10

**Table 2:** Comparison between the effect of compounds **1**, **4** and commercial drugs on platelet aggregation and plasma hemostasis, Me (25–75%).

Compound	Latent Period, % to the Monitoring	Maximum Amplitude, % to the Monitoring	Aggregation Rate, % to the Monitoring	Time to Reach MA, % to the Control	APTT, % to the Monitoring
	Collagen—Induced Aggregation	ADP—Induced Aggregation			
**1**	+5.4 (3.7–6.2) *,#	−26.4 (21.7–29.3) **,##	−15.6 (13.2–18.7) *,#	+10.1 (8.2–12.5) *	+ 9.2 (7.9–10.4) *
**4**	+13.4 (11.2–15.3) *,##	−14.5 (11.2–16.7)*	−8.5 (6.1–11.7)*	+18.7 (16.4–21.5) *,#	+5.6 (3.9–6.1) *
Acetylsalicylic acid	−2.1 (1.1–2.6)	−13.7 (10.8–16.4) *	−10.5 (7.6–12.3) *	+10.5 (8.7–13.4) *	-
Sodium heparin	-	-	-	-	20.3 (19.7–21.4) *

Note: * *p* ≤ 0.05, ** *p* ≤ 0.001—in comparison with the control; # *p* ≤ 0.05, ## *p* ≤ 0.001—in comparison with acetylsalicylic acid; data in comparison with sodium heparin are reliable (*p* ≤ 0.05); *n* = 6.

## Data Availability

Not applicable.

## Supplementary Materials

The following supporting information can be downloaded at: https://www.mdpi.com/article/10.3390/biom12111599/s1, Figure S1: Results of blind molecular docking for the interaction of compound **1** [10.1016/j.saa.2021.120638] (a), **2** (conformer A) (b), **3** (monomer A) (c), **3** (monomer B) (d), **4** (e), **5** (f) and **6** (g) with P2Y_12_.; Figure S2: Amino acid composition of P2Y_12_ binding sites with **1** [10.1016/j.saa.2021.120638] (a), **2** (conformer A) (b), **2** (conformer B) (c), **3** (monomer A) (d), **3** (monomer B) (e), **4** (f), **5** (g) and **6** (h); Figure S3: 2D diagram of **1** (a), **2** (conformer A) (b), **2** (conformer B) (c), **3** (monomer A) (d), **3** (monomer B) (e), **4** (f), **5** (g) and **6** (h) and P2Y_12_ interaction generated by Discovery Studio; Table S1: Amino acid composition of binding sites of compounds **1–6** with P2Y_12_.
