# Supplementary material for: Thioterpenoids as Potential Antithrombotic Drugs: Molecular Docking, Antiaggregant, Anticoagulant and Antioxidant Activities
Source: Biomolecules. 2022 Oct 30;12(11):1599. doi: 10.3390/biom12111599 (PMC9687754; doi:10.3390/biom12111599)
Supplement: Supplementary file 1 [file biomolecules-12-01599-s001.zip › biomolecules-1961234-supplementary.pdf]

Supplementary Materials

Thioterpenoids as Potential Antithrombotic Drugs: Molecular Docking, Antiaggregant, Anticoagulant and Antioxidant activities

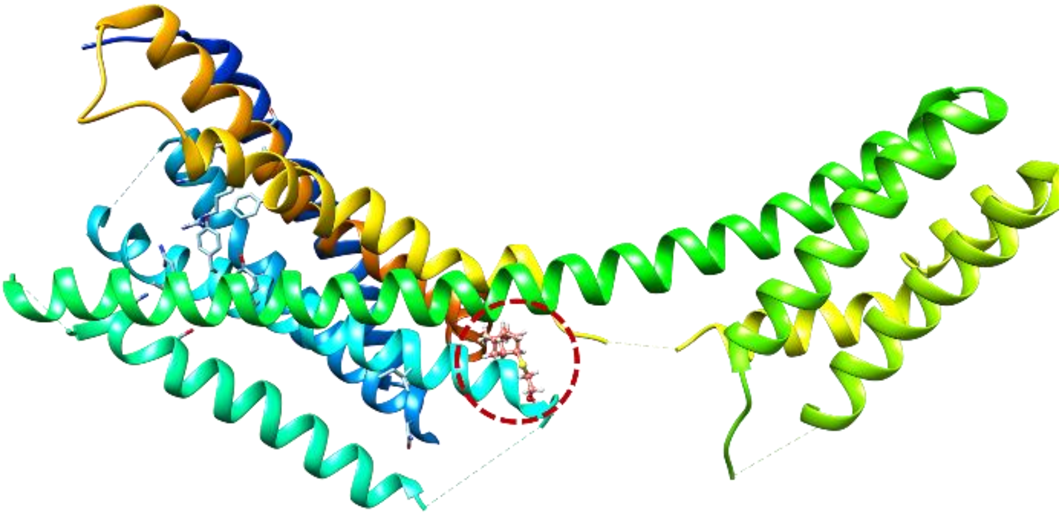

a

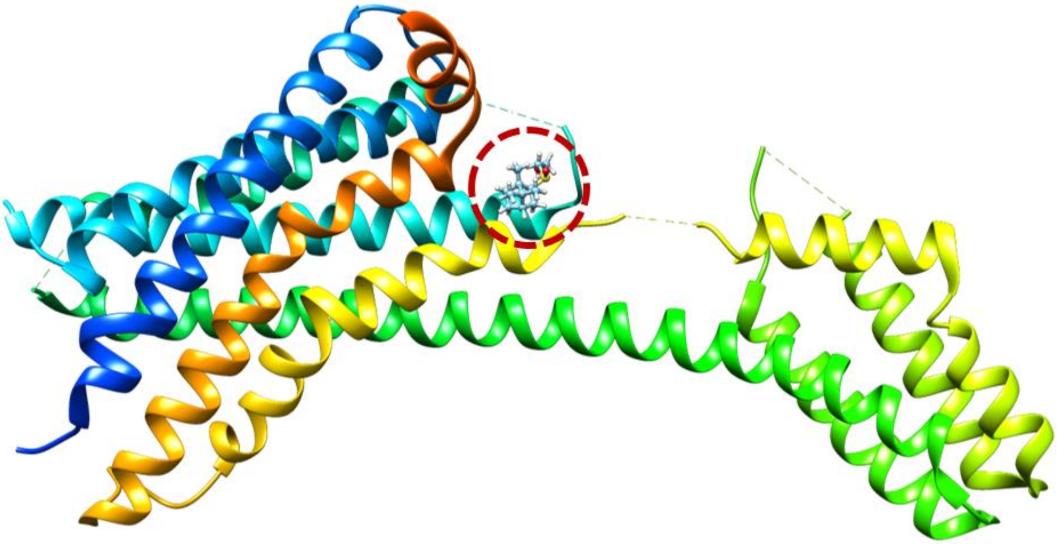

b

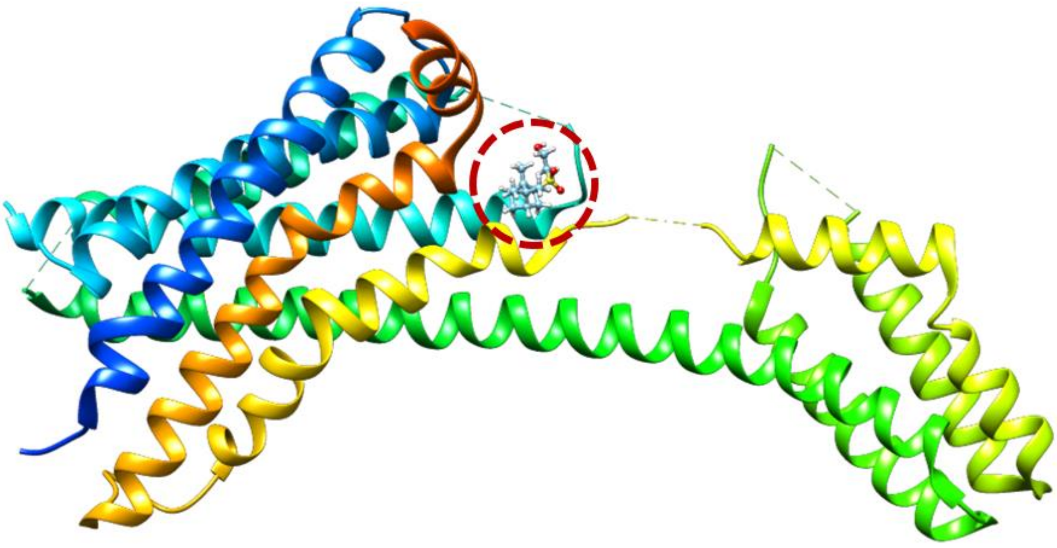

c

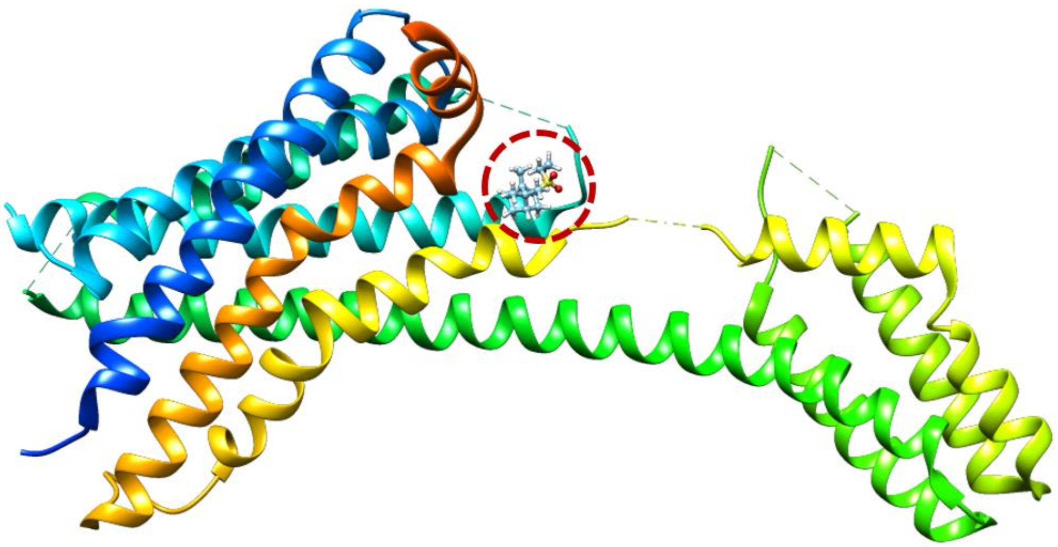

d

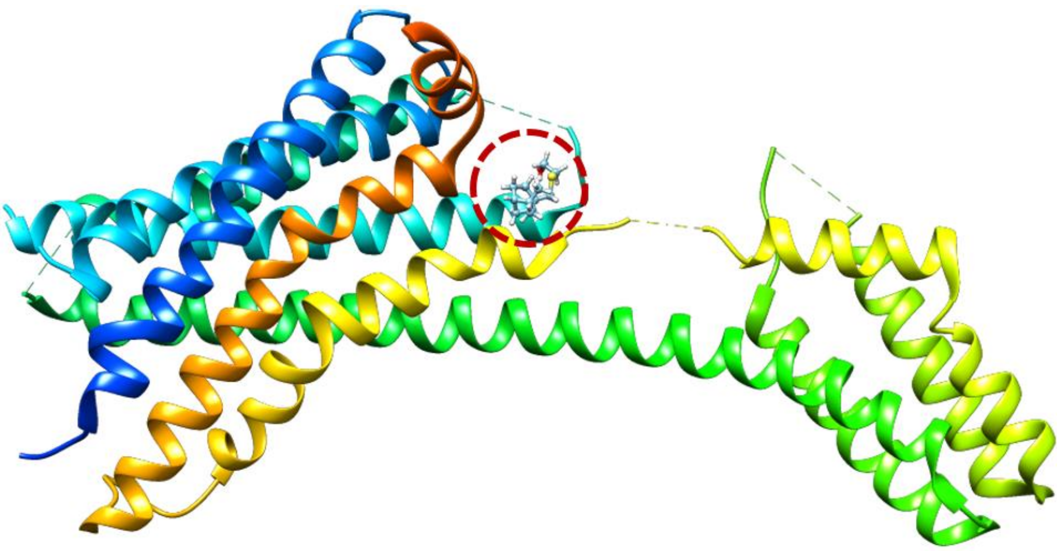

e

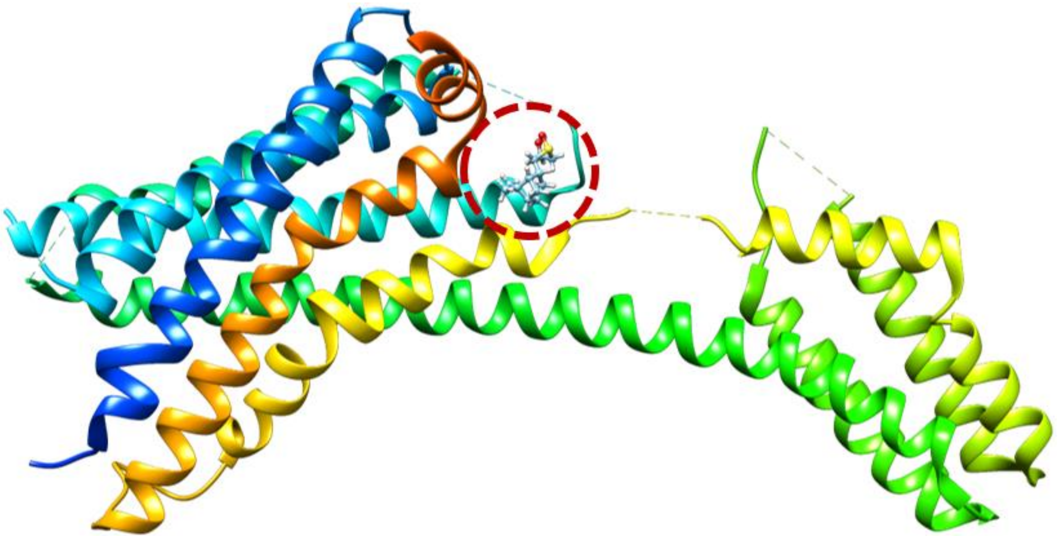

f

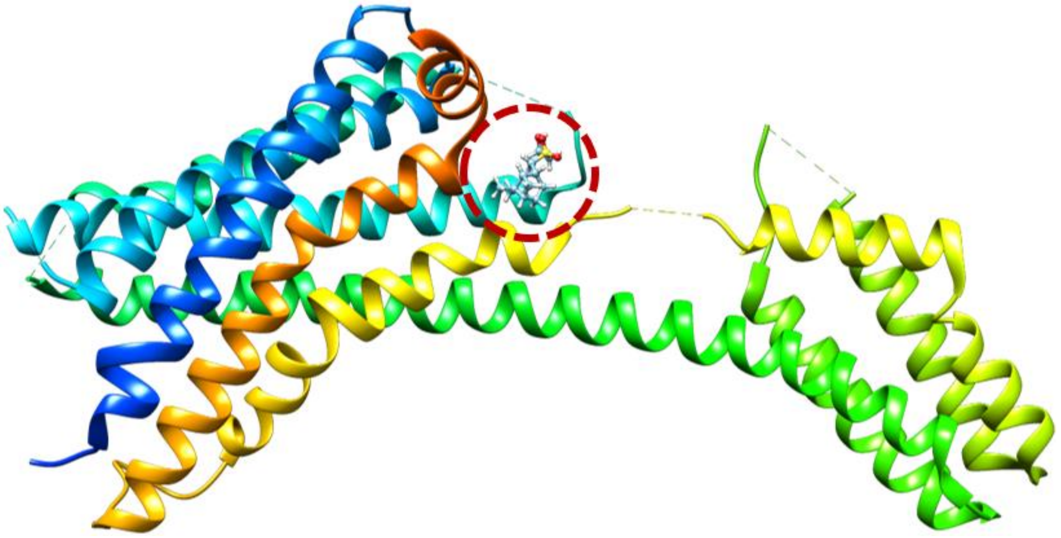

g

Figure S1. Results of blind molecular docking for the interaction of compound **1** [10.1016/j.saa.2021.120638] (*a*), **2** (conformer A) (*b*), **3** (monomer A) (*c*), **3** (monomer B) (*d*), **4** (*e*), **5** (*f*), and **6** (*g*) with P2Y12.

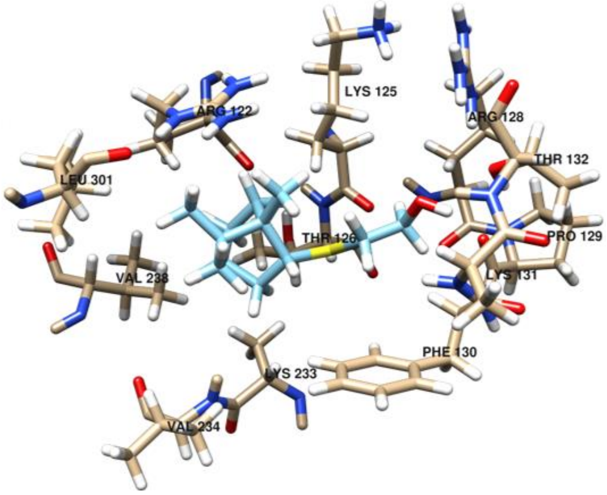

a

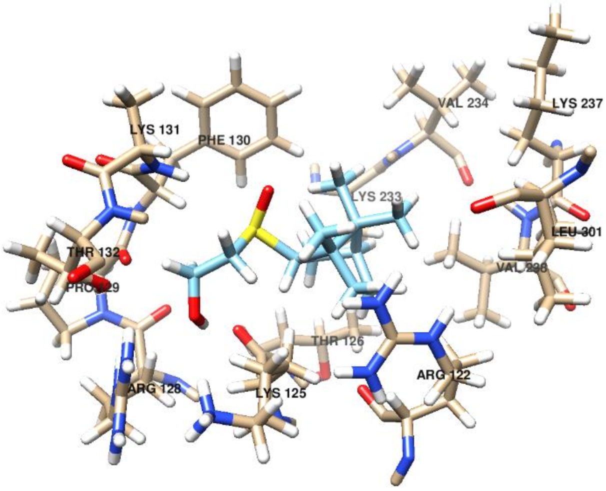

b

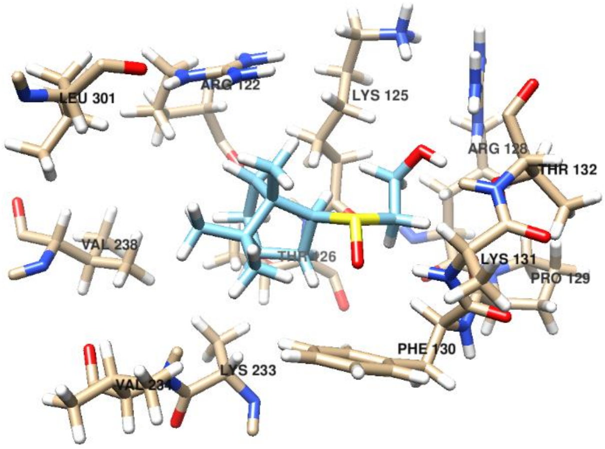

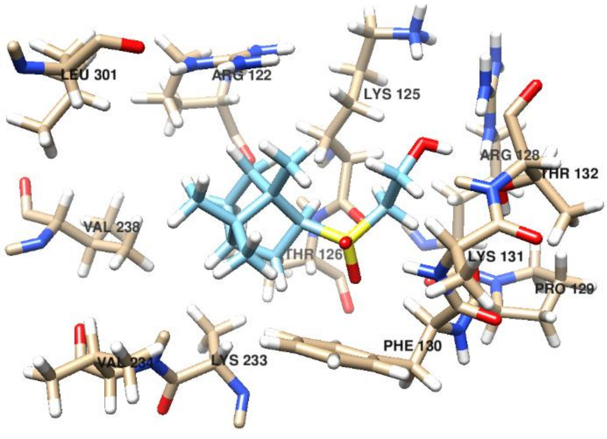

c *d*

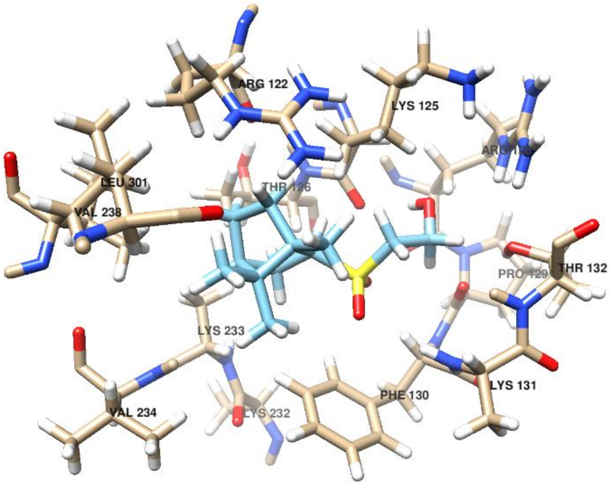

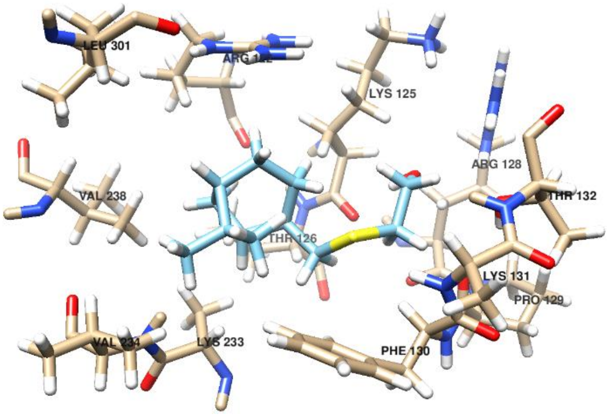

e *f*

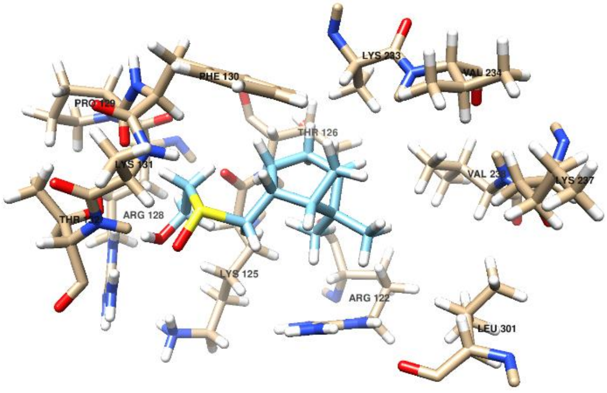

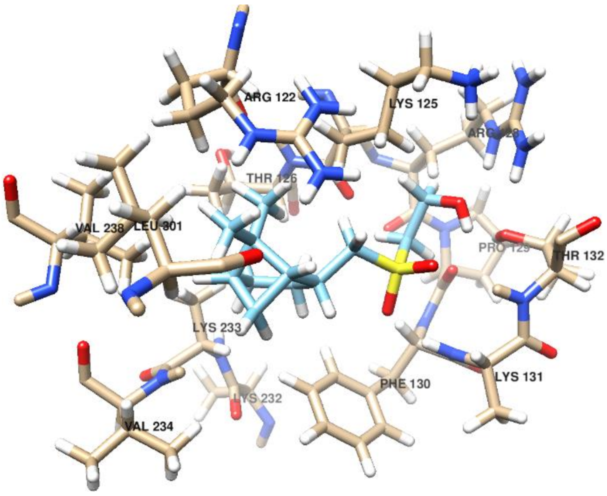

g *h*

Figure S2. Amino acid composition of P2Y12 binding sites with **1** [10.1016/j.saa.2021.120638] (*a*), **2** (conformer A) (*b*), **2** (conformer B) (*c*), **3** (monomer A) (*d*), **3** (monomer B) (*e*), **4** (*f*), **5** (*g*), and **6** (*h*).

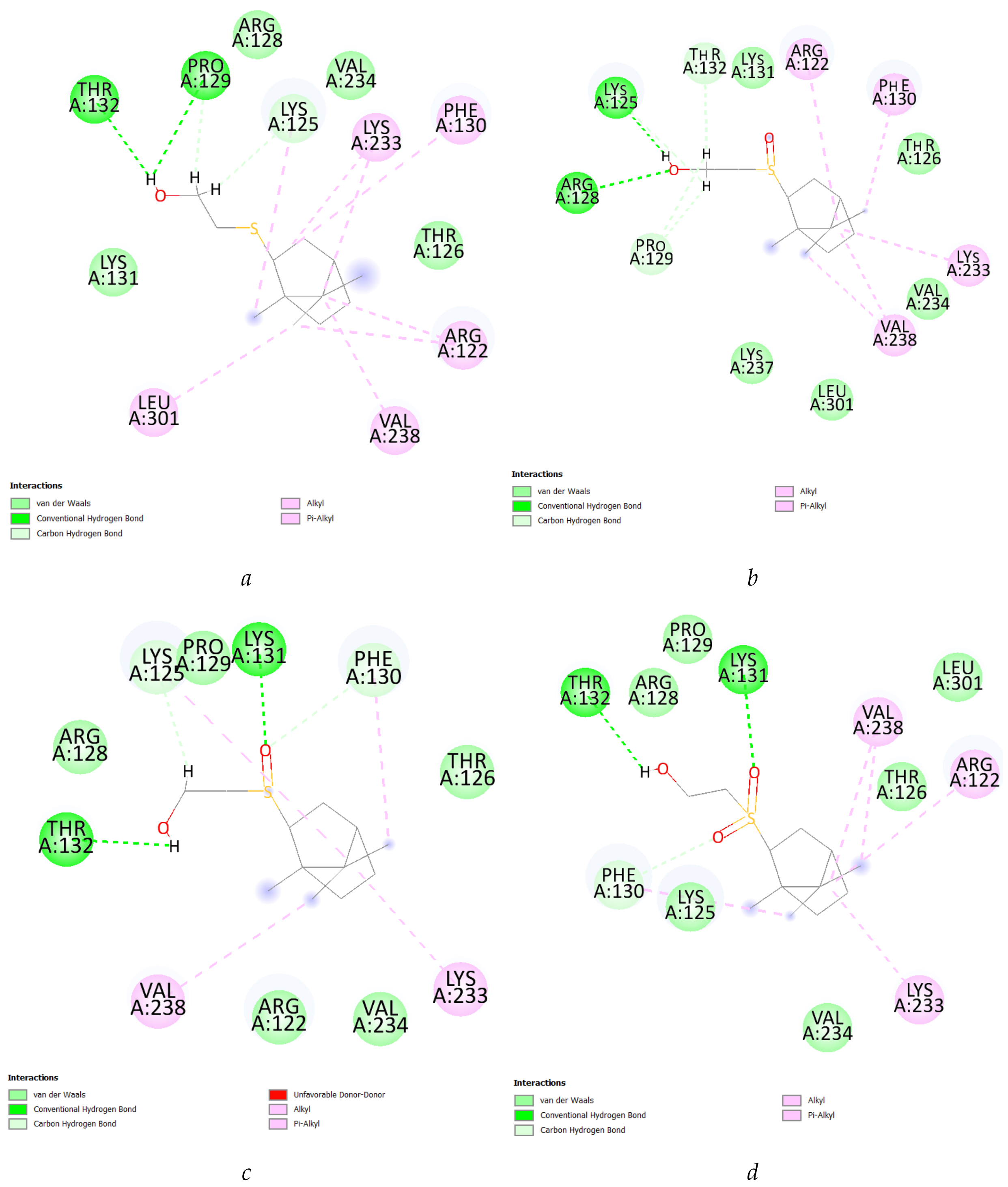

ARG A:128
PRO A:129
THR A:132
VAL A:234
LYS A:125
LYS A:233
PHE A:130
H
O
H
H
S
LYS A:131
THR A:126
ARG A:122
LEU A:301
VAL A:238
Interactions
van der Waals
Conventional Hydrogen Bond
Carbon Hydrogen Bond
Alkyl
Pi-Alkyl
a
THR A:132
LYS A:131
ARG A:122
LYS A:125
PHE A:130
O
H
H
O
S
H
THR A:126
ARG A:128
PRO A:129
LYS A:233
VAL A:234
VAL A:238
LYS A:237
LEU A:301
Interactions
van der Waals
Conventional Hydrogen Bond
Carbon Hydrogen Bond
Alkyl
Pi-Alkyl
b
LYS A:125
PRO A:129
LYS A:131
PHE A:130
ARG A:128
THR A:126
H
O
S
THR A:132
O
H
VAL A:238
ARG A:122
VAL A:234
LYS A:233
Interactions
van der Waals
Conventional Hydrogen Bond
Carbon Hydrogen Bond
Unfavorable Donor-Donor
Alkyl
Pi-Alkyl
c
PRO A:129
THR A:132
ARG A:128
LYS A:131
LEU A:301
VAL A:238
H
O
O
THR A:126
ARG A:122
S
O
PHE A:130
LYS A:125
LYS A:233
VAL A:234
Interactions
van der Waals
Conventional Hydrogen Bond
Carbon Hydrogen Bond
Alkyl
Pi-Alkyl
d

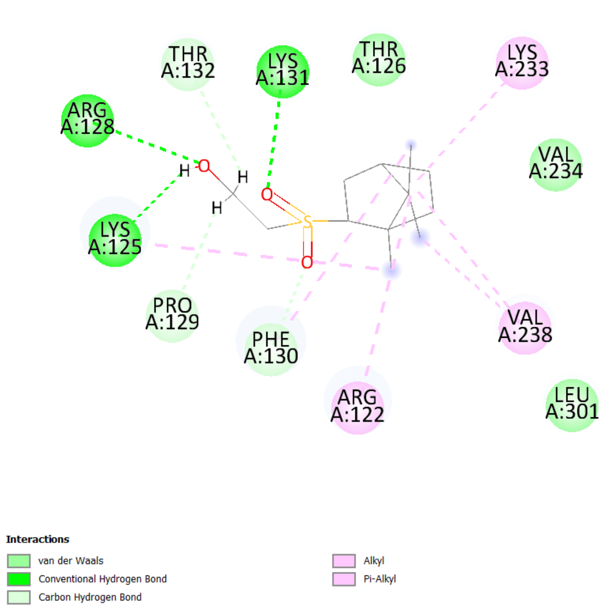

e

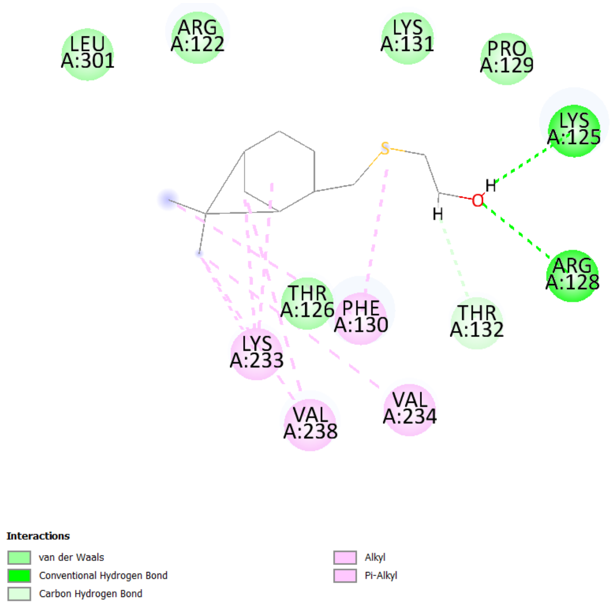

f

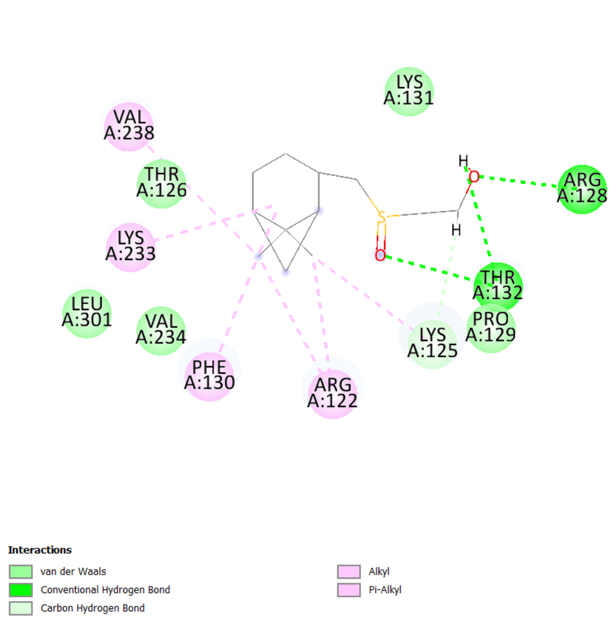

g

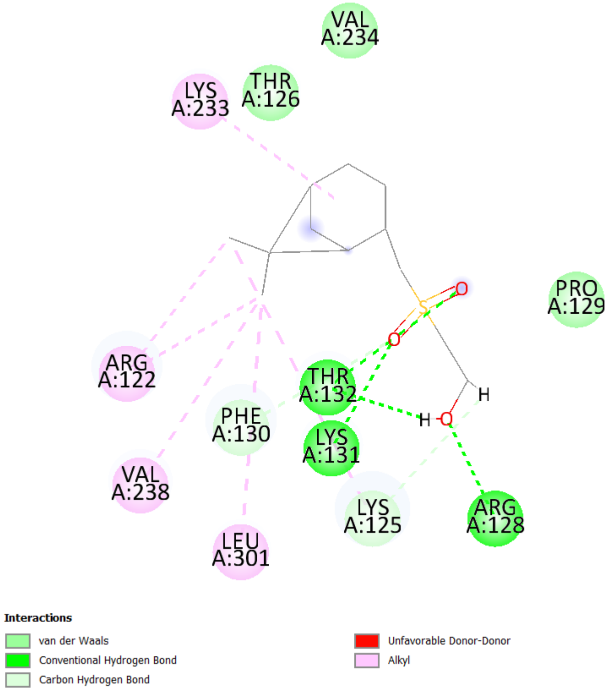

h

Figure S3. 2D diagram of **1** (*a*), **2** (conformer A) (*b*), **2** (conformer B) (*c*), **3** (monomer A) (*d*), **3** (monomer B) (*e*), **4** (*f*), **5** (*g*), and **6** (*h*) and P2Y12 interaction generated by Discovery Studio.

Table S1. Amino acid composition of binding sites of compounds **1** - **6** with $P2Y_{12}$

**Compound**	Amino acid residues
**1** [10.1016/j.saa.2021.120638]	ARG122, LYS125, THR126, ARG128, PRO129, PHE130, LYS131, THR132, LYS233, VAL234, VAL238, LEU301
**2 (conformer A)**	ARG122, LYS125, THR126, ARG128, PRO129, PHE130, LYS131, THR132, LYS233, VAL234, LYS237, VAL238, LEU301
**2 (conformer B)**	ARG122, LYS125, THR126, ARG128, PRO129, PHE130, LYS131, THR132, LYS233, VAL234, VAL238, LEU301
**3 (monomer A)**	ARG122, LYS125, THR126, ARG128, PRO129, PHE130, LYS131, THR132, LYS233, VAL234, VAL238, LEU301
**3 (monomer B)**	ARG122, LYS125, THR126, ARG128, PRO129, PHE130, LYS131, THR132, LYS232, LYS233, VAL234, VAL238, LEU301
**4**	ARG122, LYS125, THR126, ARG128, PRO129, PHE130, LYS131, THR132, LYS233, VAL234, VAL238, LEU301
**5**	ARG122, LYS125, THR126, ARG128, PRO129, PHE130, LYS131, THR132, LYS233, VAL234, LYS237, VAL238, LEU301
**6**	ARG122, LYS125, THR126, ARG128, PRO129, PHE130, LYS131, THR132, LYS232, LYS233, VAL234, VAL238, LEU301
